# Monitoring named entity recognition: the League Table

**DOI:** 10.1186/2041-1480-4-19

**Published:** 2013-09-13

**Authors:** Dietrich Rebholz-Schuhmann, Senay Kafkas, Jee-Hyub Kim, Antonio Jimeno Yepes, Ian Lewin

**Affiliations:** 1 Department of Computational Linguistics, University of Zurich, Zürich, Switzerland; 2 European Bioinformatics Institute, Wellcome Trust Genome Campus, Hinxton, Cambridge CB10 1SD, UK; 3 NICTA Victoria Research Lab, Melbourne VIC 3010, Australia; 4 Linguamatics Ltd, 324 Cambridge Science Park, Milton Road, Cambridge CB4 0WG, UK

**Keywords:** Text mining, Gold standard corpus, Evaluation, Named entity

## Abstract

**Background:**

Named entity recognition (NER) is an essential step in automatic text processing pipelines. A number of solutions have been presented and evaluated against gold standard corpora (GSC). The benchmarking against GSCs is crucial, but left to the individual researcher. Herewith we present a League Table web site, which benchmarks NER solutions against selected public GSCs, maintains a ranked list and archives the annotated corpus for future comparisons.

**Results:**

The web site enables access to the different GSCs in a standardized format (IeXML). Upon submission of the annotated corpus the user has to describe the specification of the used solution and then uploads the annotated corpus for evaluation. The performance of the system is measured against one or more GSCs and the results are then added to the web site (“League Table”). It displays currently the results from publicly available NER solutions from the Whatizit infrastructure for future comparisons.

**Conclusion:**

The League Table enables the evaluation of NER solutions in a standardized infrastructure and monitors the results long-term. For access please go to http://wwwdev.ebi.ac.uk/Rebholz-srv/calbc/assessmentGSC/. Contact: rebholz@ifi.uzh.ch.

## Background

Benchmarking components of text mining solutions against gold standard corpora (GSCs) is mandatory to achieve long-term progress in text mining [1]. The biomedical text mining community has engaged into the development of a selection of GSCs as a requirement for public competitions [2,3]. We now propose to benchmark the annotated corpora with the help of a dedicated submission site that not only benchmarks the performances, but also generates a ranked list of all-time performances (the “League Table”) and keeps hold of the submitted annotated corpora for future comparisons.

The following GSCs have been made available for the identification of gene and protein names (PGN) in the scientific literature: JNLPBA, FSUPRGE, BioCreative II and PennBioIe, and further GSCs have been prepared for chemical entities and disease mentions [4-6]. However, the evaluation of a novel NER solution against one or several GSCs is a tedious task and it is the researcher’s responsibility to perform all evaluations. The final results are reported in the corresponding scientific publication without delivering the annotated corpus to the public and without keeping track of the scores in combination with the delivered corpus.

The inclusion or exclusion of features into the NER approach decides on the performance of the solution against the GSC. It can be expected that progress in the development of NER solutions can be improved by making the annotated GSC available in combination with the system’s description and the performance measures against the used GSC. In addition, having all GSCs represented in a standard format and measuring performances through a shared submission site should reduce the error rate in all reporting. Last, the web site can act as an inventory for the annotation results related to a journal submission. Users of the site can investigate on the system descriptions and the annotation results.

Automatic evaluation has been performed as part of different challenges (e.g., LLL and BioNLP shared task), but no League Table is generated over time. The machine learning community (e.g., http://mlcomp.org) has proposed such an approach, but the GSCs for the annotation of biomedical named entities requires different evaluation methods.

Here we describe the interface of the submission site and the technology behind. A number of publicly available GSCs have been normalized into a shared representation and are available for download [7,8].

## Implementation

### Selection of GSCs

The CALBC League Table hosts GSCs for genes and proteins, for diseases and chemical entities, after serving as submission site for the CALBC challenge (Collaborative Annotation of a Large-scale Biomedical Corpus, [9]). The following GSCs for proteins and genes are accessible from the web site: (1) JNLPBA corpus (from 2004, produced from the Genia corpus), (2) BC-II (2005, test data for human gene and protein NER), (3) the PennBioIE corpus (2008, oncology), and (4) the FSU-PRGE corpus (2009, gene-regulatory events) [4-6]. All corpora deliver complete Medline abstracts as annotated documents, except the BC-II GSC which consists of individual sentences. In addition the Arizona corpus for disease annotations and the SCAI corpus for chemical entities have been normalised and uploaded to the submission site [10,11].

### Transformation to IeXML

IeXML has been used to standardize the annotations in the GSCs, which is also suitable for the alignment of the corpora. The users have to provide their annotations in the IeXML format, then upload the corpus and after a short processing time they receive the evaluation of their annotations against the corpus. The submitter is requested to have a description of the annotation solution with the uploaded annotated corpus.

Other formats have been suggested that could be used as an alternative, but would not serve the same purpose as the IeXML format. The BIO/IOB formats are very popular and have been well supported by the CoNLL challenges. The letters stand for (B)egin, (I)nside and (O)utside which represent the tag set used for marking up the different tokens of a term (B, I) and the surrounding tokens (O). Unfortunately, there is not a single standardized BIO/IOB format, i.e. different variants exist. There are leaner formats (… the_O protein_B HZF-1_I is_O …) and richer formats, which include part-of-speech information. It is possible to anticipate an XML format for BIO/IOB(<w iob="o">the</w><w iob="b">protein</w><w iob="i">HZF-1</w><w iob="o">is</w> <w iob="o">), which then could be transformed into IeXML — or even used as it is — to calculate the alignments efficiently.

Second, BIO/IOB requires that the stream of text is tokenized and usually the single tokens are delivered on separate lines. IeXML only marks and analyses the boundaries and does not consider nor evaluate the tokenisation leading to a solution whose purpose is more generic. Third, BIO/IOB – in contrast to IeXML — cannot deal with nested annotations nor with overlapping annotations, which plays an important role in biomedical text mining. For example, the phrase “left lung cancer treatment” can be annotated as a long noun phrase ("BIII"), but a more sophisticated solution would allow alternative interpretations as well which could result from the use of different terminological resources: “left/B lung/I cancer/B treatment/I” ("BIBI", a cancer treatment of an organ) and “left/B lung/I cancer/I treatment/B” ("BIIB", a treatment of a lung cancer type possibly located outside of the lungs) would both be valid solutions. In the best case the annotation solution would account for all, which cannot be achieved with BIO/IOB.

Last, BIO/IOB has so far not been used to consider the semantic type. For the sake of supporting different research communities, a transformation from BIO/IOB into IeXML is under development and will be provided in the future.

### Alignment and evaluation

The annotated corpora undergo sentence-based alignment to then achieve NE-based alignment with the NEs of the corresponding GSC using the Whatizit Finite State Automata infrastructure [12]. Alignment is performed right after submission and on the fly on a Sun Fire V40z 8-cpu opteron server with 128 GB RAM. A summary file is generated that gathers the frequency of the different error types and produces the required statistical results. Eventually, the standard statistical parameters such as precision, recall and F-measure of the annotated corpus against the GSC are calculated.

In principle, different alignments are available that produce either exact, cos98 or nested matching of the annotated entities against the pre-annotated entities in the GSC [9]. The preferred evaluation uses exact matching, since this annotation solution is the standard in public challenges. Alternative measures can be selected, such as cos98 matching and nested matching, to relax the boundary condition in the evaluation. Cos98 matching is a symmetrical measure and counts two annotations as similar, if they only have minor differences in their boundaries, i.e. the existance or lack of an extension such as a determiner or a frequently encountered term such as “protein”. Nested matching is an asymmetric measure which counts as positive, if either the GSC annotation is fully contained in the annotation of the submitted corpus, or vice versa.

In the case of BC-II, only the gene list is considered. The inclusion of the alternative gene list would lead to results that cannot be compared directly to the outcomes against the other GSCs.

## Results and discussion

The user has to select, download and annotate the GSC that fits best the user’s annotation solution. All annotations have to comply with the IeXML format for inline annotations. Standoff annotations could be used as an alternative but have proven to be less robust in challenge evaluations. The annotated corpus is submitted to the site and automatically aligned with the annotations from the GSC leading to the identification of false positive and false negative annotations. Finally the precision, recall and F-measure are determined.

The user is requested to supply a description of the annotation solution together with the annotated corpus. Currently, EBI’s publicly available annotation solutions have been applied to the GSCs and the annotated corpora have been uploaded into the League Table.

Table 1 gives an overview of the first results in the League Table. All results are sorted according to the F-measure that has been determined through the alignment of the annotated corpus against the GSC. The comparison of different PGN NER solutions has shown that their performances vary from one GSC to the next and that they achieve higher performances in the identification of PGN NER on GSCs with newer release dates [8]. Furthermore, different PGN taggers with the same F-measure performance on a given GSC can have different profiles in terms of their precision and recall performances on the GSC.

**Table 1:** The table shows the League Table for annotation solutions that have been tested against the JNLPBA GSC*

**Top performing system**								
**User**	**Reference file**	**Assessment file**	**# of**	**Precision**	**Recall**	**F-score**	**Alignment**	**Date**
			**annotations**				**type**	
jhkim	JNLPBA.Gold.xml	JNLPBA.20100730.AbnerNLPBA.xml	6142	74.70%	66.52%	70.37%	Exact	2012-02-16
jhkim	JNLPBA.Gold.xml	JNLPBA.20100730.Abner.xml	6142	61.07%	63.01%	62.03%	Exact	2012-02-16
jhkim	JNLPBA.Gold.xml	JNLPBA.20100730.chang2.xml	6142	60.27%	59.51%	59.89%	Exact	2012-02-16
jhkim	JNLPBA.Gold.xml	JNLPBA.20100730.biolexicon.xml	6142	49.17%	33.29%	39.70%	Exact	2012-02-16
jhkim	JNLPBA.Gold.xml	jnlpba.whatizitUkpmcPRGE.xml	6142	34.40%	44.45%	38.78%	Exact	2012-02-16
jhkim	JNLPBA.Gold.xml	jnlpba.swissprot70.xml	6142	39.82%	36.93%	38.32%	Exact	2012-02-16
jhkim	JNLPBA.Gold.xml	jnlpba.geneProt70.xml	6142	51.11%	30.25%	38.00%	Exact	2012-02-16
jhkim	JNLPBA.Gold.xml	JNLPBA.20100730.whatizitUkPmcGenesProteins.xml	6142	32.43%	43.87%	37.29%	Exact	2012-02-16
jhkim	JNLPBA.Gold.xml	EBI.JNLPBA.Test.xml	6142	32.53%	42.78%	36.96%	Exact	2012-01-31

^*^The same table will be shown from the League Table web interface.

The League Table approach can be applied to a variety of NE types as shown and to any selection of GSCs or silver standard corpora (SSCs). The collection of annotated corpora tagged by different tagging solutions in combination with their descriptions helps to better understand which features in the annotation solutions produce the best results.

Currently, only the U-Compare solution has been made available for comparative evaluation of annotation solutions [13]. U-Compare allows comparisons of NER solutions against publicly available tagging solutions that can be executed within U-compare, e.g., ABNER, GENIA tagger, etc., over different corpora, e.g., AImed, BioIE, and others [13]. However, U-Compare does not maintain a repository of annotated corpora and does not generate a list of performances against the GSC.

Competitions have been proposed for other tasks in computational biology, such as protein structure prediction (CASP) and the prediction of protein network representations from experimental data (DREAM) [14,15]. Furthermore, submission sites are available for generic machine-learning problems and solutions such as the MLcomp Web site [16], but this approach has not yet attracted any biomedical researchers that investigate into the semantics of the proposed task including approaches that make use of biomedical data resources. So far, the CALBC League Table is the only solution available that gathers the research community in biomedical text mining and data integration.

## Conclusions

Altogether, the CALBC League Table contributes to the development of NER solutions, since all overhead is reduced to the submission of an annotated corpus in a standardised format, and users can follow-up on their own submissions in the future. For access please go to [17]. The League Table Web interface guides all data exchange and only requires a standard Web browser for its execution.

## Competing interests

The authors declare that they have no competing interests.

## Authors’ contributions

AJY produced the core alignment engine for the assessment of submission and transformed the gold standard corpora into the standard format. AJY and JHK developed the submission site. SK and IL contributed significant efforts towards the quality control of the whole submission system. DRS supervised the work and wrote the manuscript. All authors read and approved the final manuscript.
